# Linking serum malondialdehyde with dyslipidemia in patients with metabolic syndrome

**DOI:** 10.6026/973206300211693

**Published:** 2025-06-30

**Authors:** Gadigeppa H. Chittaragi, Dileep Khubya, Veluri Ganesh

**Affiliations:** 1 Department of Medicine, Dr. N Y Tasgaonkar Institute of Medical Science, Diksal, Maharashtra, India; 2 Department of Biochemistry, Sri Siddhartha Institute of Medical Sciences, T Begur, Nelamangala, Karnataka, India; 3 Department of Biochemistry, PES University Institute of Medical Sciences and Research, Electronic City, Bengaluru, Karnataka, India

**Keywords:** Dyslipidemia, malondialdehyde (MDA), metabolic syndrome

## Abstract

Dyslipidemia and obesity is becoming an increasingly serious public health issue. Therefore, it is of interest to determine the serum levels of malondialdehyde (MDA) with dyslipidemia in patients with metabolic syndrome. The routine demographic, anthropometric and the biochemical parameters analyzed. Additionally, the degree of lipid peroxidation was measured by serum levels of malondialdehyde (MDA). The body mass index, systolic and diastolic blood pressure, fasting blood sugars, lipid profile significantly elevated in metabolic syndrome patients when compared to healthy controls. The serum MDA levels significantly elevated and correlated positively with abdominal hyperglycemia, hypertension, obesity and negatively with HDL (p=0.0001**). Thus, the early detection of people at risk for getting metabolic syndrome can be improved by measuring the MDA biomarker.

## Background:

Metabolic syndrome, a clinical disorder characterized by metabolic disturbances such as hyperglycemia, dyslipidemia, obesity and hypertension. The ages, body mass index (BMI) are risk factors of metabolic syndrome and results type 2 diabetes and cardiovascular diseases. The frequent and excessive consumption of foods and beverages high in fats and carbohydrates is one of the causes of metabolic syndrome [1-2]. The peroxidation of membrane fatty acids by Reactive Oxygen Species (ROS) is the cause of the elevated malondialdehyde (MDA) levels. The decreased levels of antioxidants also contribute to the increase of free radicals. The high levels of MDA are high indicators of metabolic syndrome [3- 4]. The lipid peroxidation (LPO) occurs when unstable molecules oxidise lipids, proteins and nucleic acids, leading to widespread cell dysfunction [5]. It starts in cell membranes when unstable free radicals steal electrons from lipids, starting a series of oxidations that cause lipid instability and the production of byproducts such MDA [6]. The oxidative stress is characterized by the presence of variables that accelerate the generation of free radicals and the loss or inability to neutralize harmful processes [7]. Therefore, it is of interest to evaluate correlation of serum malondialdehyde with dyslipidemia in patients with metabolic syndrome.

## Materials and Methods:

The present cross-sectional study included a total number of 150 metabolic syndrome patients attended the outpatients service of the Department of medicine, at Dr. N Y Tasgaonkar Institute of Medical Science, Diksal, Maharashtra were included in the present study. The metabolic syndrome patients were classified into two groups, 75 were newly diagnosed metabolic syndrome and remaining 75 metabolic syndrome patients. Seventy-five (75) ages, gender and BMI matched healthy individuals were included as controls (Table 1 - see PDF). All the participants were recruited in the study after obtaining informed consent form. The study was approved by Institutional Ethics Committee (IEC No.585 dt 09.10.2023).

## Criteria of the study:

## Inclusion criteria:

[1] Age >30 years

[2] Diagnosed to have metabolic syndrome as per NCEP-ATP III guidelines

[3] Controls without any illness

## Exclusion criteria:

[1] Patients on lipid lowering drugs,

[2] Vitamin supplements,

[3] Hormone replacement therapy and those with a history of smoking,

[4] Alcoholism, infections, abnormal renal function,

[5] Malignancy was excluded from the study.

## Sample collection:

Anthropometric data, such as height, weight and waist circumference, as well as systolic and diastolic blood pressure readings, were taken for each participant. From all the participants' peripheral venous blood samples were taken into plain (5 mL) and fluoride (2 mL) vials following an overnight fast. After that, the samples were centrifuged for 10 minutes at 3000 rpm. The separated serum (simple vial) and plasma (fluoride vial) were kept at -80°C.

## Methods:

The Fasting Blood Sugar is estimated by Glucose oxidase-peroxidase (GOD-POD) Method, Total cholesterol and Triglycerides (TGL) by Enzymatic end point colorimetric method, High density lipoprotein (HDL) cholesterol by Selective inhibition method, very low density lipoprotein (VLDL) cholesterol and low density lipoprotein (LDL) cholesterol is calculated by Friedewald Equation. Malondialdehyde (MDA) was analyzed as thiobarbituric acid reactive substances (TBARS).

## Statistical analysis:

A statistical analysis application was analysed. The Shapiro-Wilk test was used to determine the normality of the research data. If the data were normally distributed, a One-Way ANOVA would be used for analysis. In order to determine whether the distributed data was abnormal, the non-parametric Kruskal-Wallis test and the Mann-Whitney test were used for each group. The Spearman correlation was used to examine the relationship between MDA levels and Dyslipidaemia. Statistical analysis was done by using Microsoft Excel (Microsoft Corporation; Redmond) and SPSS for windows version 11.5 (SPSS, Inc., Chicago).

## Results:

Table 2 (see PDF) shows the demographic, anthropometric, biochemical and experimental parameters studied in healthy controls and patients with metabolic syndrome. There was a significance difference of age BMI, SBP and DBP between healthy controls and metabolic syndrome patients (p=0001**). The fasting blood sugars, TGL, TC and LDL levels significantly increased in metabolic syndrome patients when compared to healthy controls (p=0001**). The metabolic syndrome patients shown the significantly decreased levels of HDL when compared to healthy controls (p=0001**). Additionally the serum MDA levels significantly elevated in metabolic syndrome patients when compared to healthy controls (p=0001**). Table 3 (see PDF) shows the demographic, anthropometric, biochemical and experimental parameters studied in healthy controls and both the groups of metabolic syndrome patients. There was a significance difference of age BMI, SBP and DBP between healthy controls and both the groups of metabolic syndrome patients (p=0001**). The fasting blood sugars, TGL, TC and LDL drastically increased levels in both the groups of metabolic syndrome patients when compared to healthy controls (p=0001**). Both the groups of metabolic syndrome patients shown the drastically decreased levels of HDL when compared to healthy controls (p=0001**). Additionally the serum MDA levels drastically increased levels in both the groups of metabolic syndrome patients when compared to healthy controls (p=0001**). Table 4 (see PDF) shows the spearman's correlation analysis between serum MDA and other study variables. There was significant positive correlation between MDA and age, BMI, SBP, DBP, FBS, TGL, TC and LDL (p=0001**). Additionally, the serum MDA levels negatively correlated with HDL (p=0001**). Figure 1 (see PDF) shows the comparison of BMI in study participants. The metabolic syndrome patients have shown very high levels of BMI than the newly diagnosed metabolic syndrome patients and controls. Figure 2 (see PDF) shows the comparison of TGL in study participants. The metabolic syndrome patients have shown very high levels of TGL than the newly diagnosed metabolic syndrome patients and controls. Figure 3 (see PDF) shows the comparison of TC in study participants. The metabolic syndrome patients have shown very high levels of TC than the newly diagnosed metabolic syndrome patients and controls. Figure 4 (see PDF) shows the comparison of HDL in study participants. The metabolic syndrome patients have shown very low levels of HDL than the newly diagnosed metabolic syndrome patients and controls. Figure 5 (see PDF) shows the comparison of LDL in study participants. The metabolic syndrome patients have shown very high levels of LDL than the newly diagnosed metabolic syndrome patients and controls. Figure 6 (see PDF) shows the comparison of MDA in study participants. The metabolic syndrome patients have shown very high levels of MDA than the newly diagnosed metabolic syndrome patients and controls.

## Discussion:

Visceral obesity, atherogenic dyslipidaemia, hyperglycemia and hypertension are among the risk factors that define metabolic syndrome. Patients with metabolic syndrome are more likely to develop cardiovascular disorders, which are among the world's major causes of morbidity and mortality [8]. Numerous researches supporting the link between oxidative stress and metabolic syndrome have suggested that oxidative stress is a defining feature of metabolic syndrome and is essential to the disease's development [9-10]. The hyperglycemia and increased inflammatory markers are two examples of factors that the metabolic syndrome lowers HDL, an antioxidant and increases the formation of free radicals. When these free radicals are not neutralized by the antioxidant defense system, radicals, oxidative stress develops, which compromises the structural and functional integrity of cells [11-12]. The one of the most widely used and trustworthy biomarkers of oxidative stress is malondialdehyde, which is produced as a byproduct of lipid peroxidation as a result of free radicals [13]. The goal of this study is to assess serum levels of malondialdehyde, a biomarker of oxidative stress and compare them to the elements of metabolic syndrome. The main causes of the elevated plasma MDA concentrations in free-living persons with or at high risk for metabolic syndrome were clarified by this investigation. The higher plasma MDA concentrations were substantially correlated with altered BMI values [14]. The increased plasma MDA concentrations were also linked to increased sugar, energy intake, altered TG and glucose concentrations and the existence of metabolic syndrome. According to this perspective, lifestyle factors like inactivity and poor diet can have a noticeable impact on these markers. Whereas metabolic syndrome prevalence was greater in subjects with higher plasma MDA concentrations [15-16] is shown. The metabolic syndrome is a group of cardiovascular risk factors that include low HDL-C, high blood pressure, fasting glucose and TG and abdominal obesity. However, there is now a wealth of evidence supporting the pathogenic mechanism of elevated oxidative stress and persistent low-level inflammation in the development of metabolic syndrome-related illnesses such as atherosclerosis, endothelial dysfunction, hypertension and type II diabetes [17]. In our investigation, there was also a strong correlation between the blood level of MDA and the rise in TC, TGL, LDL, VLDL and fall in HDL, as shown in Table 3 (see PDF). Numerous investigations have found a link between dyslipidemia and elevated oxidative stress markers. The hypertriglyceridemia is proportionate to the production of tiny, dense LDL. Consequently, producing extremely all of the elements of metabolic syndrome, including reactive oxygen species, cause endothelial dysfunction, hasten atherogenesis and ultimately lead to cardiovascular disorders [18].

## Metabolic syndrome and cardiovascular disease risk:

A spectrum of cardiovascular conditions, such as microvascular dysfunction, coronary atherosclerosis and calcification, cardiac dysfunction, myocardial infarction and heart failure, are all related to metabolic syndrome [19]. Each component of the metabolic syndrome is a separate risk factor for cardiovascular disease and the combination of these risk factors increases the rates and severity of cardiovascular disease [20]. For instance, a study by reported that patients with a single metabolic syndrome component had a 2.5% risk of developing CVD in 5-years, while patients with 4 components had about a 14.9% risk of developing CVDs [21]. Compared to other risk factors of metabolic syndrome, hypertension is not only considered a major risk factor of CVD; it is regarded as a key feature of metabolic syndrome and is also attributed to about one-third of all deaths worldwide. Studies have shown that an amplified effect of metabolic syndrome is set into motion as a result of an overreaction due to overstimulation of the sympathetic nervous system (SNS) [22]. Additionally, the RAAS also indirectly raises blood pressure by acting on the water retention system, thereby causing a surge in blood pressure which is an independent and important risk factor of metabolic syndrome development. Triglycerides alone are an independent factor that contributes too many conditions that are directly and indirectly associated with metabolic syndrome and CVD [23]. Triglycerides are a risk factor for CVD events, independent of serum HDL or low-density lipoprotein (LDL) levels. Triglycerides increase the likelihood of obesity, which is a direct predisposing factor to metabolic syndrome. Therefore, triglycerides are directly associated with the development of diabetes, obesity, atherosclerotic cardiovascular disease [24]. We observed that hyperglycemia and were associated with higher plasma MDA concentrations even after adjusting for smoking and obesity. Hyperglycemia-induced oxidative stress is characterized by the presence of advanced glycation end-products (AGEs). Lipids in cell membranes can be oxidized by AGEs, which causes instability and the subsequent breakdown of LPO byproducts. In addition to being regarded as a restricted marker for evaluating total oxidative stress [25]. Higher consumption of western foods, beverages, is strongly linked to an increased risk of dyslipidemia, obesity, type II diabetes and Metabolic Syndrome, according to an elegant meta-analysis [26]. Our findings demonstrated that whereas hyperglycemia dyslipidemia and hyperadiposity can potentially lead to elevated MDA concentrations. Based on our study results confirm the theory that the dysfunctional glycated proteins, AGEs, glycooxidative stress and hyperglycaemic glycotoxicity may be the cause of lipoperoxidation and MDA formation (Figure 7 - see PDF). Lipotoxicity can also occur when lipid is pushed into organ cells, such as the liver, skeletal, heart muscle and pancreas, severely impairing their ability to function. In addition to problems in blood lipids, glucose, blood pressure, coagulation and inflammation, metabolic syndrome is a model of metabolism homeostasis breakdown that manifests as glucolipotoxicity.

## Conclusion:

High plasma MDA concentrations associated with dyslipidemia in patients with metabolic syndrome is reported. Lifestyle changes are recommended for these individuals to reduce the development of metabolic syndrome and related comorbidity. The lowering oxidative stress, dietary and exercise changes may contribute to a lower incidence of metabolic syndrome.
